# Editorial: Recent insights in vegetarian nutrition

**DOI:** 10.3389/fnut.2026.1853325

**Published:** 2026-05-14

**Authors:** Luciana Baroni, Alexey V. Galchenko, Francesca Giampieri

**Affiliations:** 1 Scientific Society for Vegetarian Nutrition, Venice, Italy; 2 Earth Philosophical Society “Melodia Vitae”, San Clemente, CA, United States; 3 Universita Politecnica delle Marche, Ancona, Italy

**Keywords:** lacto-ovo-vegetarian, plant-based diet, sustainability, vegan, vegetarian

The most investigated health and environmental effects of vegetarian diets (lacto-ovo-vegetarian and vegan) are actually well-established in current research (1–4), although some aspects remain controversial. This Research Topic offers valuable insights into some aspects of the sector, contributing to expanding current knowledge and providing some mechanistic interpretation.

Wirnitzer et al. cross-sectionally investigated the underlying motivations and lifestyle preferences related to dietary choices, and the associations between these factors and basic health behaviors in 1,350 Austrian school teachers and principals, via an online questionnaire. Health concerns (46.4%) emerged as the most significant reason for dietary choices and engagement in sports. Vegetarians represented 7.5% of the sample (5.2% lacto-ovo-vegetarians and 2.3% vegans), with a female prevalence, and their vegetable intake was significantly higher (92.9% in lacto-ovo-vegetarians and 93.5% in vegans, respectively) compared to omnivores. The Authors conclude that these educational figures, representing influential role models for their students, can contribute to public health strategies.

In their 12-week VEGPREV study (Poland), Wisniewska et al. observed a more pronounced body weight (BW) loss in obese patients, who were randomly prescribed hypocaloric vegan (100% plant-based) and EAT-Lancet Planetary Health (50% plant protein of total protein) diets, compared to those from lacto-ovo-vegetarian (70% plant protein) and Mediterranean (30% plant protein) hypocaloric diet groups. Patients who consumed a traditional hypocaloric diet following the WHO recommendations (20% plant protein) did not show any BW reduction during the study period at all.

In the two reports by Kahleova, Fischer, et al., the Authors examined whether plant-based diet indices (total plant-based dietary index-PDI, healthful-hPDI and unhealthful-uPDI) were associated with weight loss in adults with type 1 diabetes (T1DM) and in overweight adults. In a secondary analysis of a randomized trial, 58 adults with T1DM alternated between an *ad libitum* low-fat vegan diet and a portion-controlled energy-restricted omnivorous diet for 12 weeks. PDI and hPDI increased significantly in the vegan participants and were associated with weight loss, even after adjustment for changes in energy intake. Conversely, the portion-controlled group showed less change, while changes in the uPDI were not associated with weight loss.

Another secondary analysis of a randomized cross-over trial conducted by the same team involved 62 overweight adults, who alternated between Mediterranean and low-fat vegan diets for 16 weeks, with a 4-week washout period (Kahleova, Smith, et al.). Both PDI and uPDI increased during the vegan diet period, in comparison to the Mediterranean diet period, while hPDI increased during both dietary interventions. Changes in PDI and uPDI were inversely associated with changes in BW, and the association remained significant also after adjusting for changes in energy intake. Conversely, no significant association was found between changes in hPDI and BW.

The results obtained in the above three clinical trials suggest that replacing animal products with plant-based foods in a vegan diet may represent a valuable non-pharmacological intervention for weight loss, and can be beneficial in the management of overweight patients and patients with T1DM. Moreover, the effect of “unhealthy” plant-based foods on BW remains intriguing, since no negative effect was observed after their inclusion in the diet.

This aspect has been addressed by Gréa et al., who analyzed energy, nutrient contents and use of iodized salt in 964 plant-based meat substitutes on the German market. An average of 224 kcal, 13.3 g protein, 12.0 g fat, and 1.60 g salt were found per 100 g of product. Specifically, meat substitutes presented a more favorable nutritional composition compared to sausage substitutes. More than 85% of all products were labeled as vegan, and one third as organic. Nevertheless, the high heterogeneity of these products warrants a better characterization of their nutritional profiles.

In another secondary analysis of a randomized cross-over trial conducted by Kahleova, Maracine, et al. on 62 overweight adults, the dietary acid load decreased significantly within the low-fat vegan diet and was associated with weight loss, suggesting that the alkalizing effect may be an independent mechanism by which a vegan diet can promote weight loss.

Moreover, in their cohort study, Yao et al. evaluated the distinct metabolomic signatures in long-term Chinese vegetarians and omnivores, and their association with a panel of cardiometabolic risk factors. By integrating metabolomic data with detailed dietary analysis, this study identified 17 differential metabolites, 11 upregulated and six downregulated in vegetarians, some of which associated with specific plant-foods and favorably associated with obesity indices, blood pressure, lipid profiles, and insulin sensitivity. This identification of unique serum metabolomic profiles in vegetarians provides mechanistic insights into the diet-metabolite-health axis, supporting the current evidence that vegetarian diets are associated with reduced risks of cardiometabolic diseases.

Tosi et al. assessed the nutritional status and dietary intakes in a cohort of 107 newborn-mother dyads with altered biomarkers of cobalamin metabolism. Most mothers (71%) were omnivorous, 16% lacto-vegetarian, 12% lacto-ovo-vegetarian, and 1% vegan; 87% of mothers had been taking supplements during pregnancy (70.7% folic acid, 68.7% iron, 15% vitamin B_12_). Plasma vitamin B_12_ levels averaged 240 pg/ml, with supplemented mothers showing slightly higher levels compared to those who did not supplement (255.5 ± 113 vs. 231.2 ± 104 pg/ml). The Authors highlight that all dietary patterns could result in insufficient nutrient intakes, and current supplementation practices during pregnancy should be tailored.

Finally, Alcalà-Santiago et al. compared the nutritional adequacy and the environmental sustainability of four isocaloric diets—Mediterranean omnivorous, pesco-vegetarian, lacto-ovo-vegetarian, vegan—consumed for 7 days by healthy adults. No differences were found in macronutrient intakes across the four groups. All diets were low in vitamin D and iodine (except pesco-vegetarians) and in ω-3 PUFAs; the vegan diet was also low in vitamins B_12_ and calcium, but high in fiber, vitamins B_9_, C and E. Both lacto-ovo-vegetarian and vegan diets were the most sustainable models, showing a decrease in the climate change–related factors, compared to the omnivorous and pesco-vegetarian ones. By addressing the well-known critical nutrients, this study confirms that diet sustainability increases with the reduction of animal foods.

Overall, this Research Topic adds support to the beneficial effects of vegetarian diets on human and environmental health.
